# Isolation and Identification of a Tibetan Pig Porcine Epidemic Diarrhoea Virus Strain and Its Biological Effects on IPEC-J2 Cells

**DOI:** 10.3390/ijms25042200

**Published:** 2024-02-12

**Authors:** Mei Li, Meng Wang, Yao Xi, Shantong Qiu, Qiaoying Zeng, Yangyang Pan

**Affiliations:** 1 College of Veterinary Medicine, Gansu Agricultural University, Lanzhou 730070, China; lim@st.gsau.edu.cn (M.L.); wangmeng@gsau.edu.cn (M.W.); xy160117@163.com (Y.X.); qiushangtong1998@163.com (S.Q.); 2 Technology and Research Center of Gansu Province for Embryonic Engineering of Bovine and Sheep & Goat, Lanzhou 730070, China

**Keywords:** porcine epidemic diarrhoea virus, IPEC-J2, signalling pathway, apoptosis

## Abstract

Porcine epidemic diarrhoea virus (PEDV) is a coronavirus that can cause severe watery diarrhoea in piglets, with high morbidity and mortality rates, seriously hindering the healthy development of the global swine industry. In this study, we isolated a strain of PEDV from Tibetan pigs and named it CH/GS/2022. Subsequently, we screened the apoptosis signals of PEDV-infected IPEC-J2 cells and studied the correlation between apoptosis signals and cell apoptosis. The results showed that different infections of PEDV induced different degrees of apoptosis in cells, and PEDV-induced cell apoptosis was dose-dependent. We then detected the expression of the p53, p38, JNK, Bax, and Bcl-2 genes in the apoptosis signal pathway. The results showed that 24 h after PEDV infection, the expression of the p53, p38, JNK, and Bax genes in IPEC-J2 cells increased significantly, while the expression of the Bcl-2 gene decreased significantly (*p* < 0.05). Subsequently, we used Western blot to detect the protein levels of these five genes, and the results showed that PEDV infection upregulated the expression of p53, p38, JNK, and Bax proteins (*p* < 0.05) while downregulating the expression of Bcl-2 protein (*p* < 0.05). Thus, it was initially inferred that PEDV infection could regulate cell apoptosis by activating the p53, p38, and JNK signalling pathways. Finally, we further investigated the apoptosis of the cells through the use of inhibitors. The results indicated that the p53 inhibitor Pifithrin-α has a significant inhibitory effect on the expression of the p53 protein after PEDV infection and can reverse the expression levels of Bax and Bcl-2 proteins. This suggested that p53 is involved in PEDV-induced cell apoptosis. Similarly, the p38 MAPK inhibitor SB203580 has an inhibitory effect on the expression of the p38 protein and can reverse the expression levels of Bax and Bcl-2 proteins. This suggested that p38 is also involved in PEDV-induced cell apoptosis. On the other hand, the JNK inhibitor SP600125 has no inhibitory effect on the expression of the JNK protein after PEDV infection, but the expression levels of Bax and Bcl-2 proteins have changed. Furthermore, it is noteworthy that SP600125 can inhibit the activity of apoptotic proteins but not their levels, resulting in reduced cell apoptosis. These preliminary results indicated that JNK may be involved in PEDV-induced IPEC-J2 cell apoptosis.

## 1. Introduction

Porcine epidemic diarrhoea (PED) is an acute, high-contact infectious disease of the intestinal tract of pigs caused by porcine epidemic diarrhoea virus (PEDV) with watery diarrhoea, vomiting, dehydration, and anorexia as clinical features [1]. PEDV belongs to the family *Coronaviridae*, the genus Coronavirus, and is susceptible to different strains and stages of infection, with clinical symptoms varying depending on the age of onset [2]. In the late 1970s, the virus first appeared in England, followed by outbreaks in Europe and Asia with the classical strain CV777 [3]. The spread of PEDs has led to major economic problems in the global pork industry [4].

The PEDV particles have a generally spherical and polygonal shape, ranging from 95 to 190 nm (average diameter 130 nm), an outer capsid membrane, and petal-like cilia, which are 18–23 nm in length [5,6]. PEDV is a positive-sense single-stranded RNA virus with a genome size of approximately 28 kb, a cap structure at the 5’ end of the genome, a poly(A) tail at the 3’ end of the genome, and a total of seven open reading frames (ORFs) [1,7]. Four structural proteins can be directly translated from nucleic acid through transcription, namely fibrillar(S), envelope(E), membrane(M), and nucleocapsid(N), in addition to 16 non-structural proteins (nsp1-nsp16) and auxiliary proteins ORFs [8]. PEDV is mainly transmitted via the faecal–oral route, with large numbers of viral particles found in infected pigs’ intestinal tracts [9]. It is possible to protect against PEDV using inactivated or attenuated vaccines; however, it can fail if there is an immune deficiency or new mutant strains, especially with the emergence of mutated strains in Asian countries after 2010 [10,11]. As the main target cells for PEDV, intestinal epithelial cells play a critical role in protecting the body against microorganism infection [12]. The villous tissue of the small intestine suffers visible atrophy due to acute necrosis and exfoliation caused by PEDV infection [13]. When the structural integrity of the small intestinal villi is compromised, their immune barrier effect is suppressed, thereby amplifying viral infection; thus, a pig infected with PEDV showed a reduction in intestinal villus height and a decrease in transdermal cellular resistance [14,15]. Specific intracellular signalling networks are activated as a result of the cellular perception of external stimuli, such as viral and bacterial infections, which in turn alter cellular biological properties, with the cell cycle, apoptosis, and proliferation of cells being some of them [16,17].

There has been a significant improvement in our understanding of the molecular virology, epidemiology, and vaccination of PEDV since the emergence of mutant strains. Additionally, there have been studies on the molecular mechanisms of the PEDV infection response; for example, Xu et al. demonstrated that porcine epidemic diarrhoea virus infection induces Vero cell apoptosis through the reactive oxygen species (ROS)/p53 signalling pathway [18], Yang et al. confirmed that porcine epidemic diarrhoea virus induces vero cell apoptosis via the p53-PUMA signalling pathway [19], and Shen et al. proved that PEDV infection blocks the cell cycle and induces apoptosis in pig intestinal epithelial cells [17]. However, the research on the molecular mechanisms of host cell response to PEDV infection is insufficient, especially in terms of pathological changes in host cell biological characteristics. Additionally, Vero cells, which are monkey kidney cells of African green origin, have been used as the basis for most investigations on the pathophysiology and molecular processes of PEDV-infected host cells. In recent years, several studies have identified the porcine small intestinal epithelial cell line (IPEC-J2) as a suitable model system for PEDV infection [20,21,22]. In this study, we examined the processes of cell–virus interactions, isolated and characterised putative PEDV samples, and examined the effects of PEDV infection on the biological characteristics of IPEC-J2 cells.

## 2. Results

### 2.1. Microscopic Observation of the Effect of Viruses on Cell Morphology

**Figure 1:**
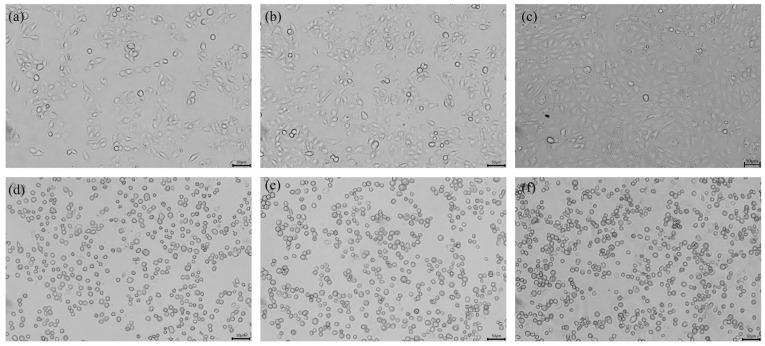
**Inverted microscopy assays of PEDV-infected IPEC-J2 cells (200×).** (**a**) Mock-infected: 12 h; (**b**) mock-infected: 18 h; (**c**) mock-infected: 24 h; (**d**) PEDV-infected: 12 h; (**e**) PEDV-infected: 18 h; and (**f**) PEDV-infected: 24 h. Cells were grown on 24-well plates and incubated with PEDV for different times; CPEs appeared to start from 12 h and became more evident at 18 h and 24 h compared with the negative control infection.

The infection of PEDV typically induces typical cytopathic effects (CPEs) in cells, such as swelling, aggregation, shedding, and vacuole formation, ultimately leading to cell death [23]. To confirm whether the suspected infected tissue virus fluid causes CPEs, we first observed the morphological changes of the infected IPEC-J2 cells. The results are shown in Figure 1. When the cells inoculated with the suspected virus fluid were passed to the fifth generation, cell pathology was observed. The control group cells were neatly arranged, uniform in size, and had clear cell edges, while the infected group began to show cell pathology at 12 h, with cell clustering, swelling, and rounding. Compared with the control group, the cell shape appeared more three-dimensional. After 18 h and 24 h of infection, CPEs became more pronounced, with over 80% of the cells displaying CPEs, cell shedding and vacuolisation further intensified, and some cells were suspended in the culture medium (Figure 1).

### 2.2. Real Time PCR Confirmed the Identity of the Suspected Virus as PEDV

**Figure 2:**
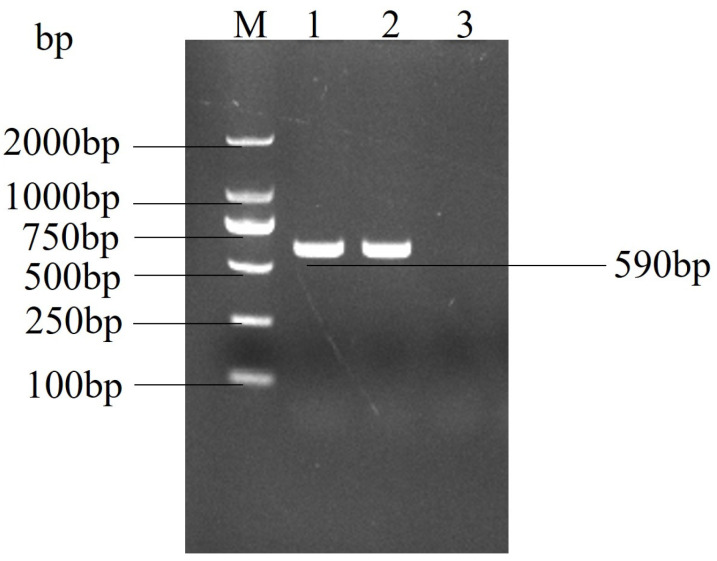
**Agarose gel electrophoresis results of real-time PCR amplification of the PEDV.** M: DL 2000 DNA Marker; 1: CH/GS/2022; 2: positive control (CV777); and 3: negative control (the template is water).

Total RNA was extracted and reverse transcribed from the cell culture and subjected to real-time PCR. The PCR product was detected by 1.0% agarose gel electrophoresis, and a target band of approximately 590 bp was amplified (Figure 2). The results showed a specific band compared to the control cell product, which was sequenced to confirm that the isolated virus was porcine epidemic diarrhoea virus and was designated as strain PEDV-GS22.

### 2.3. Indirect Immunofluorescence Identified the Virus as PEDV

**Figure 3:**
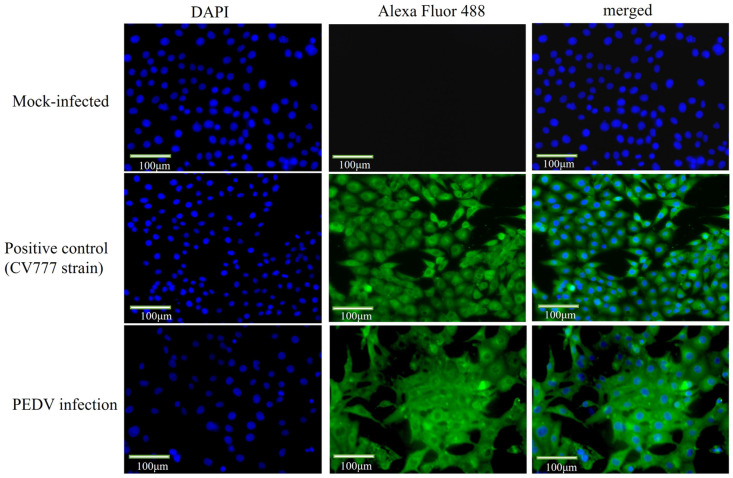
**Immunofluorescence results for virus identification (400×).** Immunofluorescence assay further identified the virus as PEDV. IPEC-J2 cells were grown on coverslips in 12-well plates and infected with PEDV of 1 MOI. Cells were fixed and incubated with PEDV N protein monoclonal antibody (1:200) and Alexa Flour488 in the infected group, and picture acquisition was performed after infection; mock-infected: normal cells; positive control: CV777.

To further confirm that the virus is PEDV, we added a PEDV N protein monoclonal antibody and performed IFA testing. Compared with the control cells, the specific green fluorescence was observed on the IPEC-J2 cells infected with PEDV (Figure 3), indicating that the isolated virus was able to interact with the monoclonal antibody against PEDV protein and further confirming the success of PEDV isolation.

### 2.4. Observation of Viral Particles in Ultra-Thin Sections of Cells

**Figure 4:**
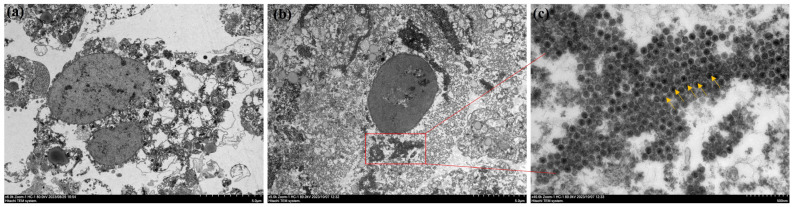
**Electron microscopic observations of ultra-thin sections of PEDV-infected IPEC-J2 cell cultures.** (**a**) Mock-infected: normal cells (5.0 μm); (**b**) infected group (5.0 μm); (**c**) infected group: yellow arrows point to particles of PEDV (500 nm).

Observation of ultra-thin sections of IPEC-J2 cells showed that no viral particles were found in the mock-infected group, whereas a large number of PEDV particles were present in the infected group as compared to the mock-infected group. IPEC-J2 cells are derived from the small intestine of piglets, and after PEDV infection in piglets, the virus content was highest in the small intestine, causing necrosis and shedding of intestinal cells. A large number of virus particles were detected inside the cells, leading to blurred cell morphology and difficult-to-recognise structures [24] (Figure 4).

### 2.5. Results of Phylogenetic Evolutionary Analysis of CH/GS/2022 Isolate Genes

**Figure 5:**
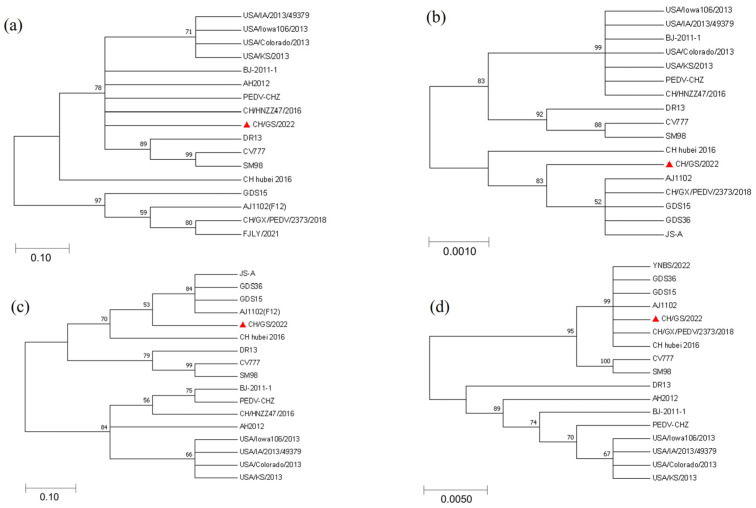
**Phylogenetic evolutionary analysis of the genes of the CH/GS/2022 isolate.** (**a**) Nucleotide genetic evolution analysis of S gene of CH/GS/2022 strain; (**b**) nucleotide genetic evolution analysis of N gene of CH/GS/2022 strain; (**c**) nucleotide genetic evolution analysis of M gene of CH/GS/2022 strain; (**d**) nucleotide genetic evolution analysis of ORF3 gene of CH/GS/2022 strain.

The amino acid sequences of S, N, M, and ORF3 proteins of the CH/GS/2022 strain isolated in this experiment were compared with the sequences of 16 representative PEDV strains from GenBank using MEGA7.0 sequence analysis software. Phylogenetic tree analysis was carried out using the proximity method. The results of the S protein comparison showed that strain CH/GS/2022 was more closely related to the strains BJ-2011-1, AH2012, PEDV-CHZ, and CH/HNZZ47/2016; the results of the N protein comparison showed that strain CH/GS/2022 was more closely related to strains JS-A, GDS36, GDS15, AJ1102(F12), and CH/GX/PEDV/2373/2018; the results of M protein comparison showed that strain CH/GS/2022 was more closely related to strain JS-A, GDS36, GDS15, and AJ1102(F12); and the alignment results of ORF3 protein showed that the CH/GS/2022 strain is closely related to strains YNBS0222, GDS15, GDS36, AJ1102, CH/GX/PEDV/2373/2018, and CH-Hubei-2016. It is noteworthy that, based on the genetic analysis of N protein and M protein, this strain of virus belongs to a different lineage than other strains, which may be related to virus evolution and genetic diversity. Solid triangles mark the CH/GS/2022 strain isolated in this experiment (Figure 5).

### 2.6. Apoptosis Is Induced by PEDV Infection in a Dose-Dependent Manner

**Figure 6:**
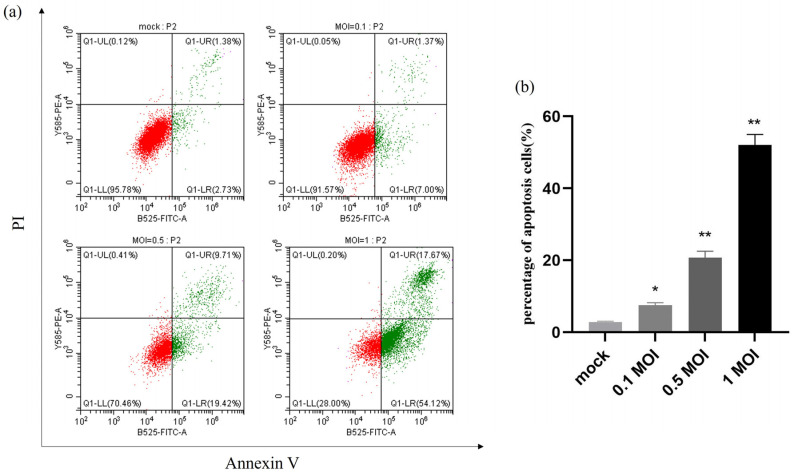
**PEDV infection induces apoptosis in a dose-dependent manner.** (**a**) Mock and infected IPEC-J2 cells at MOI of 0.1, 0.5, and 1 were collected and stained with Annexin-V-FITC and PI at 24 h post-infection and then analysed by flow cytometry. (**b**) Percentages of Annexin-V-FITC-positive cells from gated cells. Results are representative of three independent experiments. Data are represented as mean ± SD, n = 3. (* *p* < 0.05; ** *p* < 0.01).

The apoptotic rate of PEDV-infected IPEC-J2 cells at MOIs of 0.1, 0.5, and 1 was precisely determined by flow cytometry analysis of mock and infected cells that were collected 24 h after infection. The results showed that the apoptosis rate was significantly higher after PEDV infection at different doses compared with the control group, and the apoptosis rate reached 42% at 1 MOI, indicating that the apoptosis after PEDV infection was dose-dependent with the virus (Figure 6).

### 2.7. qRT-PCR to Detect the Relative Expression of Genes at Various Stages of PEDV Infection of IPEC-J2 Cells

**Figure 7:**
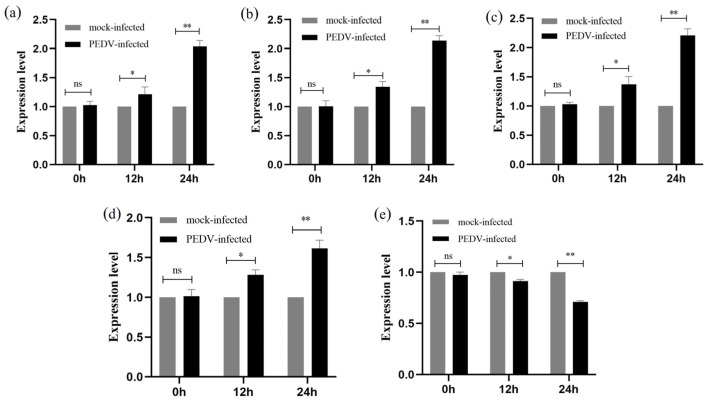
**Relative expression of five genes in IPEC-J2 cells after PEDV infection by qRT-PCR.** (**a**–**e**) are the gene expression of p53, p38, JNK, Bax, and Bcl-2 after PEDV infection of IPEC-J2 cells, respectively. All results were normalised to the mock-infected. Results are representative of three independent experiments. Data are represented as mean ± SD, n = 3 (ns: *p* > 0.05; * *p* < 0.05; ** *p* < 0.01).

By using qRT-PCR, it was possible to measure the relative expression of five genes (p53, p38, JNK, Bax, and Bcl-2) in IPEC-J2 cells that were PEDV-free and infected with PEDV (MOI = 1) at 0 h, 12 h, and 24 h. All five genes were persistently expressed in IPEC-J2 cells that had been infected with PEDV for 12 or 24 h, as shown in Figure 7. The findings of the qRT-PCR demonstrated that when PEDV infected IPEC-J2 for 12 h, the expression of the five genes, p53, p38, JNK, Bax, and Bcl-2, did not change substantially in comparison to the PEDV-uninfected group (*p* > 0.05). The expression of p53, p38, JNK, and Bax genes was significantly increased (*p* < 0.01), while the expression of the Bcl-2 gene was significantly decreased (*p* < 0.01) at 24 h of PEDV infection in IPEC-J2 cells (Figure 7).

### 2.8. Expression of Activation-Associated Signalling Proteins by PEDV Infection on IPEC-J2 Cells

**Figure 8:**
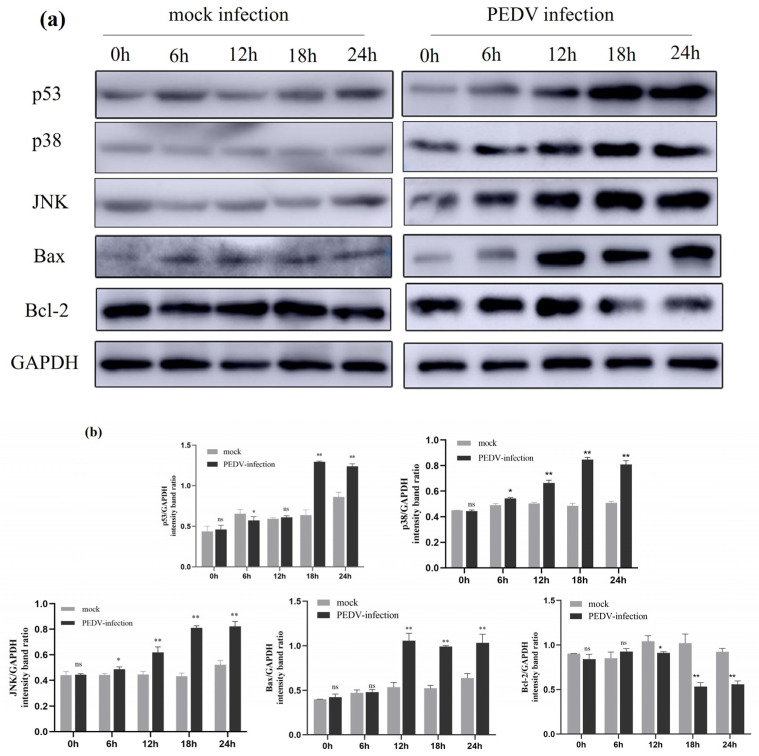
**Effect of PEDV infection on p53, p38, and JNK signalling pathway-related protein expression shown by Western blotting.** (**a**) Effect of PEDV infection on the expression of apoptosis-related proteins shown by Western blotting. (**b**) The intensity band ratio of Western blot and all results normalised to GAPDH. Results are representative of three independent experiments. Data are represented as mean ± SD, n = 3 (ns: *p* > 0.05; *, *p* < 0.05; **, *p* < 0.01).

The expression of apoptosis-related proteins in uninfected PEDV and PEDV-infected (MOI = 1) IPEC-J2 cells at different times (0 h, 6 h, 12 h, 18 h, and 24 h) was detected using Western blotting. As shown in the figure, compared to the uninfected group, the expression levels of p53, p38, and JNK proteins started to change at 6 h post-PEDV infection (*p* < 0.05). At 12 h post-PEDV infection, the expression levels of p38, JNK, and Bax proteins significantly increased (*p* < 0.01), and at 18 h post-infection, the expression level of p53 protein also significantly increased (*p* < 0.01). After 12 h of PEDV infection, the expression level of Bcl-2 protein decreased (*p* < 0.05), and at 18 h and 24 h post-infection, the decrease in Bcl-2 protein expression was more marked (*p* < 0.01). The results indicate that PEDV infection activates the expression of related signalling in IPEC-J2 cells (Figure 8).

### 2.9. ROS Accumulate in PEDV-Infected IPEC-J2 Cells in a Time-Dependent Manner

**Figure 9:**
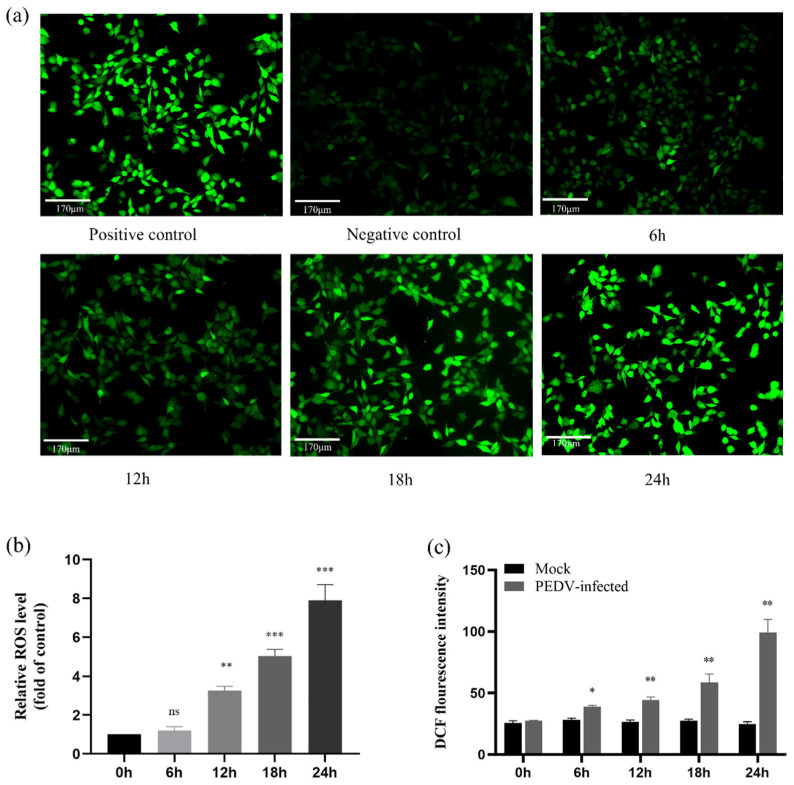
**Detection of intracellular ROS levels by PEDV infection of IPEC-J2 cells.** (**a**) The intensity of cellular fluorescence after different periods of PEDV infection of IPEC-J2 cells using fluorescence microscopy (200×); positive control: contains the positive control reagent Rosup; and negative group: normal cells. (**b**) Relative fluorescence intensity of cellular DCF after PEDV infection of IPEC-J2 cells (ns: *p* > 0.05; ** *p* < 0.05; *** *p* < 0.01). (**c**) DCF fluorescence intensity of cells after PEDV infection of IPEC-J2 cells (All results normalised to the mock group) (* *p* < 0.05; ** *p* < 0.01).

By triggering the mitochondria-mediated apoptotic pathway, ROS contributes significantly to apoptosis [25,26]. We employed DCFH-DA to measure the level of ROS in PEDV-infected (MOI = 1) cells using fluorescence microscopy and multifunctional enzyme markers to measure the cellular fluorescence intensity and absorbance values to analyse the degree of ROS expression during PEDV-induced apoptosis. The results showed that the fluorescence intensity of PEDV-infected cells increased with time, especially at 24 h post-infection, compared with the control group. ROS levels were further analysed and quantified in PEDV-infected cells using the histogram method. As shown in the figure, ROS were significantly accumulated at different time intervals with time dependence (*p* < 0.05) compared with the control group. The data results suggest that PEDV infection can cause ROS accumulation in IPEC-J2 cells (Figure 9). 

### 2.10. Effect of Inhibition of p53 Signalling Pathway on Apoptosis and Apoptotic Proteins

**Figure 10:**
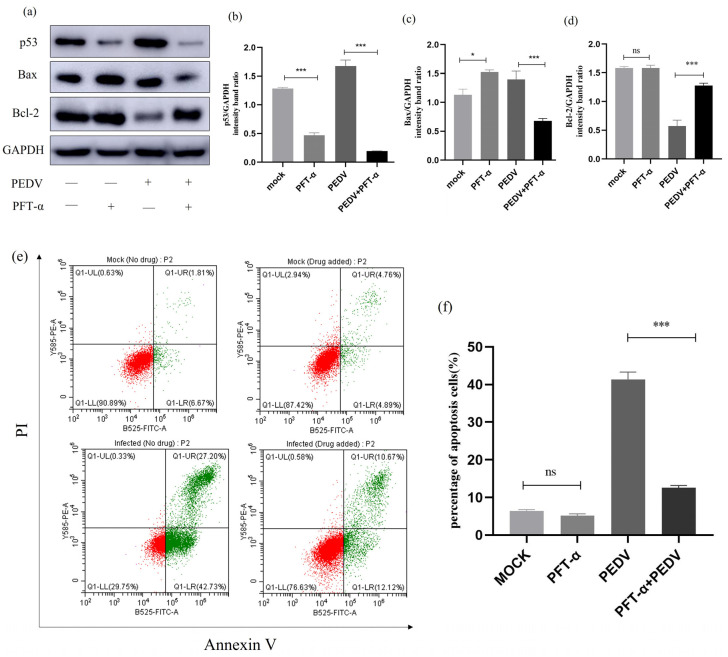
**PFT- α-treated p53 signalling pathway-related protein expression.** (**a**) Effect of the p53-specific inhibitor (PFT-α) on the total amount of p53 and the expression of Bcl-2 and Bax. IPEC-J2 cells were infected with PEDV for 24 h in the presence or absence of PFT-α (10μM). Cells were collected and subjected to Western blot analysis. (**b**–**d**) Representative densitometry of p53, Bax, and Bcl-2 was calculated after being normalised to GAPDH using Image J. (**e**) Mock and infected IPEC-J2 cells in the presence or absence of PFT-α were collected and stained with Annexin-V-FITC and PI at 24 h post-infection and then analysed by flow cytometry. (**f**) Percentages of Annexin-V-FITC-positive cells from gated cells. Results are representative of three independent experiments. Data are represented as mean ± SD, n = 3 (ns: *p* > 0.05; *, *p* < 0.05; ***, *p* < 0.01).

To investigate the relationship between p53 and cell apoptosis during PEDV infection, we treated PEDV-infected (MOI = 1) IPEC-J2 cells with p53-specific inhibitor Pifithrin-α (PFT-α) and observed changes in the expression levels of relevant proteins. As shown in Figure 10a,b, PFT-α can effectively inhibit the expression of the p53 protein. Next, we studied the effect of PFT-α treatment on the protein expression of Bax and Bcl-2. Western blot analysis revealed that after PFT-α treatment of PEDV-infected IPEC-J2 cells, the Bax protein level was significantly downregulated, while the Bcl-2 protein level was upregulated. Furthermore, flow cytometry was used to detect changes in the apoptosis rate of cells after PFT-α treatment. The results showed that compared to the PEDV-infected group, the apoptosis rate of cells induced by PEDV was significantly reduced after PFT-α treatment. The results indicate that the addition of a p53 inhibitor to PEDV-infected cells leads to a decrease in the rate of apoptosis, indicating that p53 is involved in PEDV-induced cell apoptosis (Figure 10).

### 2.11. Effects of p38 MAPK Inhibition on Apoptosis and Apoptotic Proteins

**Figure 11:**
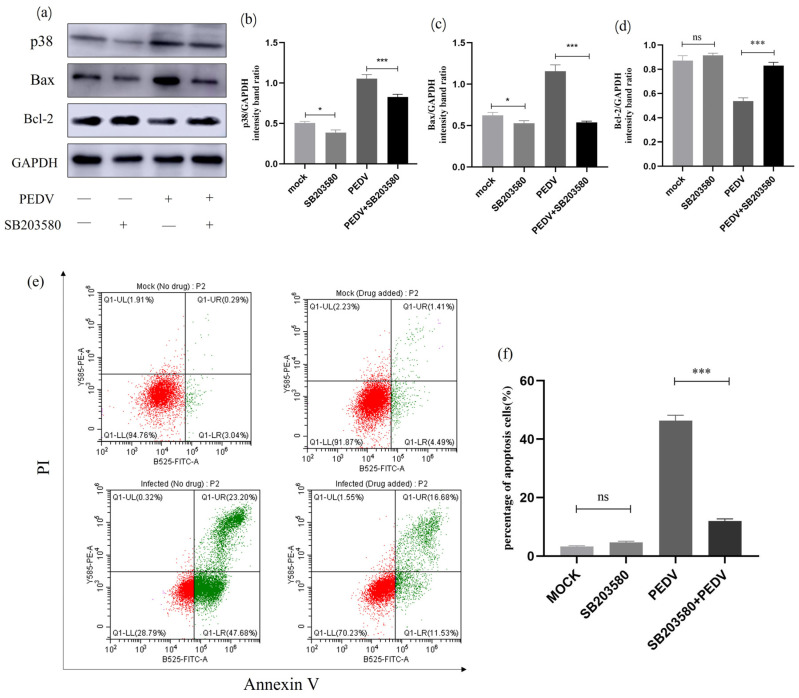
**SB203580-treated p38 MAPK signalling pathway-related protein expression.** (**a**) Effect of the p38-specific inhibitor (SB203580) on the total amount of p38 and the expression of Bcl-2 and Bax. IPEC-J2 cells were infected with PEDV for 24 h in the presence or absence of SB203580 (10μM). Cells were collected and subjected to Western blot analysis. (**b**–**d**) Representative densitometry of p38, Bax, and Bcl-2 was calculated after being normalised to GAPDH using Image J. (**e**) Mock and infected IPEC-J2 cells in the presence or absence of SB203580 were collected and stained with Annexin-V-FITC and PI at 24 h post-infection and then analysed by flow cytometry. (**f**) Percentages of Annexin-V-FITC-positive cells from gated cells. Results are representative of three independent experiments. Data are represented as mean ± SD, n = 3 (ns: *p* > 0.05; *, *p* < 0.05; ***, *p* < 0.01).

To investigate the relationship between p38 MAPK and cell apoptosis during PEDV infection, we treated PEDV-infected (MOI = 1) IPEC-J2 cells with p38-specific inhibitor SB203580 and observed changes in the expression levels of relevant proteins. As shown in Figure 11, Western blot analysis revealed that the treatment of cells with SB203580 resulted in decreased expression levels of p38 protein. Additionally, IPEC-J2 cells infected with PEDV and treated with SB203580 showed a significant reduction in p38 protein expression while also exhibiting a clear downregulation of Bax protein levels and upregulation of Bcl-2 protein levels. Flow cytometry was further used to detect changes in the apoptosis rate of SB203580-treated cells. It can be concluded that SB203580 treatment significantly reduced PEDV-induced apoptosis in IEC-J2 cells. These results indicate that the addition of p38 inhibitor in PEDV-infected cells leads to a decrease in apoptotic rate, suggesting the involvement of p38 in PEDV-induced cellular apoptosis (Figure 11).

### 2.12. Effect of JNK Inhibition on Apoptosis and Apoptotic Proteins

**Figure 12:**
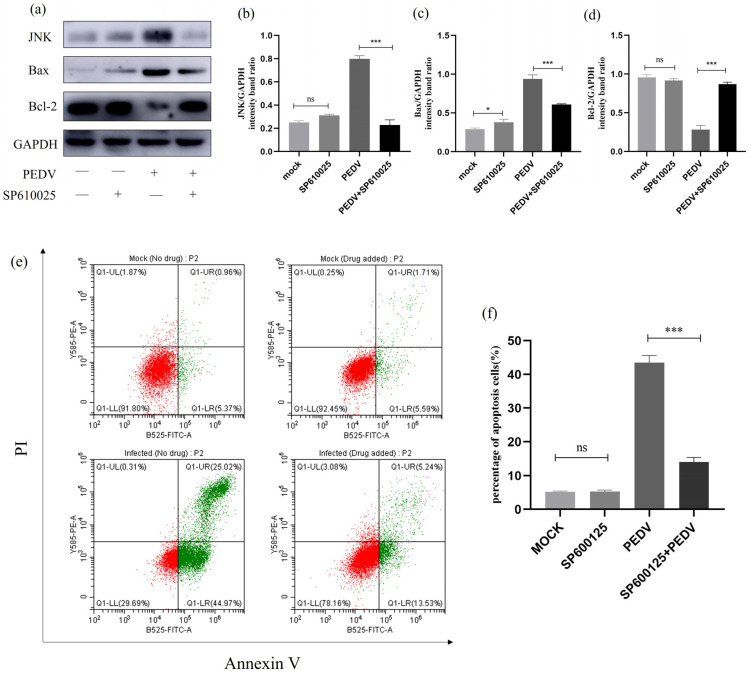
**SP600125 -treated JNK signalling pathway-related protein expression.** (**a**) Effect of the JNK-specific inhibitor (SP600125) on the total amount of JNK and the expression of Bcl-2 and Bax. IPEC-J2 cells were infected with PEDV for 24 h in the presence or absence of SP600125 (10μM). Cells were collected and subjected to Western blot analysis. (**b**–**d**) Representative densitometry of JNK, Bax, and Bcl-2 was calculated after being normalised to GAPDH using Image J. (**e**) Mock and infected IPEC-J2 cells in the presence or absence of SP600125 were collected and stained with Annexin-V-FITC and PI at 24 h post-infection and then analysed by flow cytometry. (**f**) Percentages of Annexin-V-FITC-positive cells from gated cells. Results are representative of three independent experiments. Data are represented as mean ± SD, n = 3 (ns: *p* > 0.05; *, *p* < 0.05; ***, *p* < 0.01).

To investigate the relationship between JNK and cell apoptosis during PEDV infection, we treated PEDV-infected (MOI = 1) IPEC-J2 cells with JNK-specific inhibitor SP600125 and observed changes in the expression levels of relevant proteins. As shown in Figure 12, Western blot analysis revealed that there was no change in JNK protein levels after treating the cells with SP610025. However, after treating the PEDV-infected IPEC-J2 cells with SP610025, there was a significant decrease in JNK protein expression levels, accompanied by a significant downregulation of Bax protein levels and an upregulation of Bcl-2 protein levels. Flow cytometry was further used to detect changes in the apoptosis rate of SP610025-treated cells. It can be concluded that SP610025 treatment significantly reduced PEDV-induced apoptosis in IPEC-J2 cells. These results indicate that JNK may be associated with PEDV-induced apoptosis in IPEC-J2 cells, but further research is needed for confirmation (Figure 12).

### 2.13. No Inhibitory Effect of Inhibitors on Cell Activity

**Figure 13:**
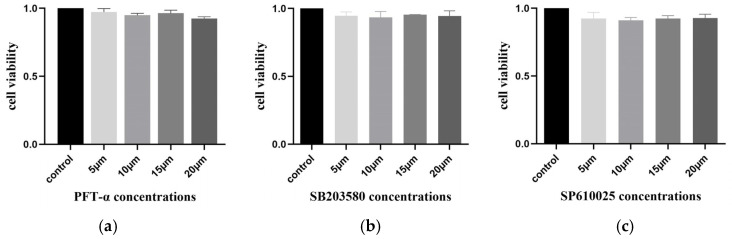
**The effect of different concentrations of inhibitors on IPEC-J2 cell viability.** (**a**) Cell viability of IPEC-J2 after the effect of different concentrations of PFT-α; control: normal cells. (**b**) Cell viability of IPEC-J2 after the effect of different concentrations of SB203580; control: normal cells. (**c**) Cell viability of IPEC-J2 after the effect of different concentrations of SP610025; control: normal cells (ns: *p* > 0.05).

To eliminate the influence of the inhibitors PFT-α, SB203580, and SP610025 on cell viability, the MTT method was used to detect the cell viability of IPEC-J2 cells after 24 h of treatment with the inhibitors. The results showed that none of the three inhibitors had an impact on cell viability after 24 h (Figure 13).

## 3. Discussion

Piglets are susceptible to PEDV, an alpha coronavirus that causes severe intestinal diarrhoea. China’s discovery of a highly pathogenic G2b subtype mutant strain in October 2010 was followed by a pandemic in the United States in 2013, which spread to Canada and Mexico. PEDV has become prevalent worldwide, causing huge economic losses to the world’s pig industry and seriously affecting the world’s development of the pig farming industry [27,28]. Further investigation is necessary as the pathophysiology of PEDV remains unclear. The genetically regulated, autonomous, and orderly death of cells is known as apoptosis. It is regulated by pro- and anti-apoptotic cytokines and is brought on by a variety of extracellular or intracellular stimuli [29,30]. Many viruses can actively induce apoptosis and thereby promote viral replication, facilitating the release and spread of viral progeny to neighbouring cells [31,32]. This pro-apoptotic event is one of the cytolytic properties of viral infections that can lead to cytopathic effects in cells cultured in vitro and exert pathogenic effects in cells, such as cellular damage, tissue injury, and disease in vivo [33,34].

For the regulation of apoptosis, the activation of many cell signalling pathways is involved [35,36]. A multitude of internal processes and biological processes, including cell cycle regulation, differentiation, apoptosis, and inflammatory responses in particular cell types under varying stressors, are carried out via the p53 signalling pathway [37,38]. Prior research on coronaviruses has revealed that PEDV produces cell cycle arrest in the G0/G1 phase via a p53-dependent route [39] and that PVP infection causes PK-15 apoptosis by activating the p53 and mitochondria-mediated apoptosis pathways [40]. Members of the Bcl-2 family play a very important role during apoptotic responses [41,42]. Based on their roles, members of the Bcl-2 family can be divided into three groups: pro-apoptotic proteins Bax, pro-apoptotic activators like NOXA and PUMA, and anti-apoptotic proteins Bcl-2 [43,44]. Bcl-2 binds to Bax and interacts to prevent mitochondrial pore formation, thereby inhibiting the execution of cell apoptosis [45,46]. In this study, we found that PEDV infection can promote the expression of p53 in IPEC-J2 cells at 18 h post-infection. Significant changes in the expression levels of Bax and Bcl-2 proteins were observed after PEDV infection, and flow cytometry analysis confirmed that the use of a p53 inhibitor significantly reduced the apoptosis rate of the cells, indicating that inhibiting p53 can reverse PEDV-induced cell apoptosis.

ROS are secondary products of oxidative metabolism and consist mainly of non-radical substances such as oxygen radicals, OH^-^ radicals, and some peroxides such as H_2_O_2_ and HNOO^−^ [47]. Evidence from a growing number of studies suggests that ROS are associated with apoptosis induced by many viral infections [48]. In the present study, we used the DCFH-DA assay to examine the fluorescence intensity as well as ROS expression levels in PEDV-infected cells to determine whether ROS accumulation is associated with apoptosis. The fluorescence intensity of PEDV-infected cells was dramatically elevated, as was the level of ROS expression, compared to the negative control group, indicating that ROS were implicated in PEDV-induced apoptosis in IPEC-J2 cells.

MAPK is a family of serine/threonine kinases capable of responding to intracellular signals generated by various stimuli. Oxidative stress-dependent activation of MAPK is involved in the induction of apoptosis [49,50,51]. Furthermore, we also discovered that PEDV infection induces the activation of p38 MAPK and SAPK/JNK, maintaining high levels of protein expression. In inhibitor experiments, it was observed that SB203580 treatment significantly reduced P38 protein expression, and compared to the PEDV infection group, Bax and Bcl-2 protein levels underwent significant changes. However, SP600125 had no significant effect on JNK protein expression, requiring further research. Flow cytometry confirmed a significant decrease in the cell apoptosis rate after inhibitor usage. The results indicated the involvement of p38 MAPK in the PEDV-induced apoptosis of IPEC-J2 cells and provided initial insights into the potential involvement of JNK in the PEDV-induced apoptosis of IPEC-J2 cells, which requires further study for confirmation. In addition, it is worth noting that in the inhibitor experiment, the results of flow cytometry showed that compared to the control group, the number of necrotic cells in the inhibitor group increased. However, after inhibitor treatment, the number of necrotic cells in infected cells significantly decreased. The occurrence of this phenomenon was unexpected, and we speculate that there may be cellular necroptosis. However, further research is needed to confirm this finding.

In summary, this study successfully isolated a strain of porcine epidemic diarrhoea virus (PEDV) and demonstrated the involvement of ROS and p53 in the PEDV-induced apoptosis of IPEC-J2 cells. Additionally, it provides initial insights into the potential involvement of p38 MAPK and JNK in the process. These findings offer further data support for understanding the pathogenesis of PEDV.

## 4. Materials and Methods

### 4.1. Cells

IPEC-J2 cells (CVCL-2246) were grown in DMEM with 10% inactivated foetal bovine serum (FBS; PAN-Biotech, Aidenbach, Germany) and 1% penicillin/streptomycin (Invitrogen, Waltham, MA, USA) and incubated at 37 °C with 5% CO_2_ [52].

### 4.2. Inhibitors and Antibodies

Sigma (Sigma-Aldrich, Shanghai, China) provided the p53 inhibitor Pifithrin-α (PFT-α), while Merck (Merck KGaA, Darmstad, Germany) provided the p38 MAPK inhibitor SB203580 and the SAPK/JNK inhibitor SP600125. Anti-p53, p38 MAPK, JNK/SAPK, Bax, Bcl-2, and GAPDH antibodies were purchased from Abmart (Abmart Shanghai Co., Ltd., Shanghai, China).

### 4.3. Disease Material Collection and Handling

The small intestine tissues previously collected aseptically were chopped and placed in a sterile mortar. Liquid nitrogen was added to grind the tissues into a powdered form, followed by the addition of a DMEM high-glucose culture medium containing gentamicin. The mixture was subjected to three repeated freeze–thaw cycles and centrifuged at 12,000× *g* for 10 min, and the supernatant was collected and filtered through a 0.22 μm filter membrane to prepare the virus solution, which was stored at −80 °C for later use.

### 4.4. Virus Isolation and Culture

A total of 1 mL of the prepared virus solution was inoculated into IPEC-J2 cells cultured at 80% confluence, and the cells were inoculated for 2 h in a 37 °C, 5% CO_2_ cell culture incubator. The virus solution was poured off, and 7 mL of the DMEM high-glucose medium (1% FBS and trypsin at a final concentration of 5 ng/mL) was added to the cells for daily observation of cell morphology changes. The viruses were collected at 72 h, then the viruses were frozen and thawed three times, and the supernatants were filtered with a 0.22 μm filter membrane for 10 min to obtain the virus solution F1. The virus was sequentially cultured and isolated in this way until cellular lesions were observed, and the harvested viral solution was stored at −80 °C [53].

### 4.5. Virus Purification by Density Gradient Centrifugation

After carefully balancing the virus-containing solution, it was subjected to ultracentrifugation at 20,000 rpm for 2 h. The supernatant was then discarded, and the precipitate was dissolved in STE buffer. Subsequently, the virus-containing sample was added to the ultracentrifuge tube, followed by the sequential addition of 30%, 45%, and 60% sucrose. Ultrahigh-speed centrifugation at 30,000 rpm for 2.5 h was performed, and the transparent bands between the sucrose gradients were carefully extracted to obtain the purified virus solution, which was stored at −80 °C for future use.

### 4.6. Detection of Viral Titres by the TCID50 Method

The cell density was adjusted to 1 × 10^4^ cells/mL, and the cell suspension was sequentially added into 96-well plates to 100 μL/well and incubated at 37 °C with 5% CO_2_ for 24 h. When the cell density reached 90%, the virus solution to be tested was diluted in multiplicity (10^−1^–10^−10^), the virus solution was added into 96-well plates according to the dilution in order, and 8 replicates were set for each group. A negative control group (containing only normal cells) was also set up. After 90 min of virus infection, 100 μL/well of the DMEM high-sugar medium (containing 1% FBS and trypsin at a final concentration of 5 ng/mL) was added to the plates, the cells were incubated continuously for 5–7 days, and the changes in the cells were observed every day. The TCID50 was calculated according to the Reed–Muench method (Reed and Muench 1938).

### 4.7. Identification of Viruses

#### 4.7.1. Real-Time PCR Assay

Total RNA was extracted by the Trizol method and reverse transcribed according to the PrimeScript™ Real-time Master Mix kit (Takara, Beijing, China). We took 1 μL each of PEDV-F and PEDV-R primers, 1 μL of the reverse transcription product, 12.5 μL of 2×Mix, and 9.5 μL of ddH_2_O for a total volume of 25 μL. The reaction conditions were 95 °C for 5 min, 95 °C for 30 s, 58 °C for 30 s, 72 °C for 1 min, 35 cycles, and 72 °C for 10 min. The PCR products were identified by 1.0% agarose gel electrophoresis. PEDV-specific primer sequences are as follows (Forward: 3′-TAGGACTCGTACTGAGGGTGT-5′; Reverse: 3′-CTATTTTCGCCCTTGGGAATT-5′).

#### 4.7.2. Indirect Immunofluorescence for PEDV

IPEC-J2 cells were infected with PEDV virus solution for 1 h, then the virus solution was discarded, and the cells were observed until slight cytopathic lesions appeared. The cells were fixed with 4% paraformaldehyde for 15 min, permeabilised with 0.5% Triton X-100 for 20 min at room temperature, blocked with 5% bovine serum albumin (BSA) for 1h, and incubated with monoclonal antibody against PEDV N protein (1:200) overnight. The cells were incubated with goat anti-mouse Alexa Flour 488 fluorescent secondary antibody (1:100) at 37 °C for 1 h. The cells were washed with phosphate-buffered saline (PBS) for 3 min three times; then, the nuclei of the cells were stained with DAPI dropwise and incubated for 5 min, and then the cells were washed well with PBS 5 times. Treated cells were visually examined using a laser scanning confocal microscope (Leica Microsystems, Wetzlar, Germany).

#### 4.7.3. Cell Ultra-Thin Section Observation

IPEC-J2 cells were inoculated with PEDV virus solution, the cells were observed until lesions appeared, and then the virus solution was discarded. Additionally, 2.5% glutaraldehyde was added to fix the cells for 10 min away from light, the cells were scraped with a cell scraper in the same direction and centrifuged at 1000 rpm/min for 5 min, and the fixative was discarded. The cells were washed with PBS, 1% osmium tetroxide was added to the cells for 1 h, they were washed four times in PBS, and then the cells were resin-embedded with ethanol after gradient dehydration, stained, and observed under a transmission electron microscope (RuliTEM, Tokyo, Japan).

#### 4.7.4. Analysis of PEDV Genetic Evolution

The RNA of IPEC-J2 cells inoculated with PEDV was used as a template, referencing the literature, and specific primers were synthesised for the PEDV S, N, M, and ORF3 genes [54,55]. The amplified products were sent to Jin Wei Zhi Bio-technology Co. Ltd. (Shanghai, China) for sequencing, and then the gene sequences of the isolates of the S gene, the M gene, the N gene, and the ORF3 gene were compared with the gene sequences of the 25 representative strains of PEDV in the database of GenBank. The sequences of S, M, N, and ORF3 genes of the isolates were compared with those of 25 representative strains of PEDV in the GenBank database (Supplementary Materials), the sequence homology between the strains was analysed using DNA STAR 6.0, and phylogenetic analysis was carried out by using Mega 11 software.

### 4.8. Apoptosis Assay

The Annexin V-FITC Apoptosis Kit (BioVision, Inc., Milpitas, CA, USA) was used for apoptosis detection according to the manufacturer’s instructions. Cells were washed twice with PBS and then resuspended in 500 μL of a binding buffer, followed by the addition of 5 μL of Annexin V-FITC and 5 μL of PI. After incubation for 30 min at room temperature and protection from light, 20,000 cells were collected, and the percentage of positive cells was analysed by flow cytometry (Becton Dickinson, Franklin Lakes, NJ, USA).

### 4.9. Real-Time Quantitative PCR to Detect the Expression of Related Genes

Using GAPDH as an internal reference, the experiment was conducted according to the instructions of the ChamQ SYBR qPCR Master Mix kit (Vazyme, CH). A total of 10 μL of 2×ChamQ Green; primer1 and primer2, each 0.4 μL; and cDNA (diluted 1:10), 5 μL, were taken for the experiment. The amplification conditions were set as follows: 95 °C for 10 s, 60 °C for 30 s, and 72 °C for 20 s. Subsequently, the expressions of p53, p38, JNK, Bax, and Bcl-2 were detected using the Recycle 96 System (Light Cycler 96), relevant primers information is in Table 1.

### 4.10. Western Blot Assay Analysis

Cells were collected, washed with pre-cooled PBS, and then treated with RIPA lysis buffer containing 1 mM phenylmethylsulfonyl fluoride (PMSF). Protein concentration was determined using a BCA protein assay reagent (Pierce, Appleton, WI, USA). Equal amounts of proteins were loaded and electrophoresed on 8–12% sodium dodecyl sulfate-polyacrylamide gel electrophoresis (SDS-PAGE). Subsequently, proteins were transferred onto polyvinylidene difluoride (PVDF) membranes (Millipore Corp, Atlanta, GA, USA). The membranes were closed in Tris-buffered saline containing 5% skimmed milk in 0.1% Tween 20 (TBST) for 2 h at room temperature, and then we diluted the primary antibody (1:1000) in TBST and incubated it overnight at 4 °C. We incubated the HRP-conjugated secondary antibody (1:5000) in TBST at room temperature for 2 h. Protein expression was detected using an ECL reagent (GE Healthcare, Chicago, IL, USA).

### 4.11. ROS Detection

At various intervals, cells were infected with PEDV, and the cells were then harvested. The cells were rinsed three times with PBS and then treated with 10 μM DCFH-DA for 30 min at 37 °C before being rinsed three times with brand-new basal media devoid of serum. Under a fluorescence microscope, the cells’ fluorescence was monitored, and a fluorescent multifunctional enzyme labelling device (Molecular Devices, San Jose, CA, USA) was used to measure the absorbance of the cells.

### 4.12. Inhibitor Assay

P53-specific inhibitor PFT-α was dissolved in dimethyl sulfoxide at a concentration of 10 mM, and p38MAPK-specific inhibitor SB203580 and JNK-specific inhibitor SP600125 were dissolved in dimethyl sulfoxide at a concentration of 20 mM and stored at −20 °C. The inhibitor stock solution was diluted with DMEM to a working concentration of 10 μM. Six-well plates were used, and cells were washed with PBS three times before adding 1 mL of the inhibitor working solution to each well for pretreatment for 1 h. PEDV was then inoculated, the cells were incubated in a CO_2_ incubator for 90 min and washed with PBS twice, and 1 mL of inhibitor working solution was added to each well. The cells then continued to be cultured in the incubator for 24 h and were collected at the specified time for the detection of relevant indicators.

### 4.13. MTT Assay

The cell concentration was adjusted to 5 × 10^4^/mL, and then the cells were inoculated into 96-well plates at 100 μL/well. After the cell density reached 80%, different concentrations of inhibitors PFT-α, SB203580, and SP600125 were added and incubated for 24 h. Meanwhile, a negative control group was set up (containing only normal cells). Then, 20 μL of an MTT labelling reagent was added to each well and incubated at 37 °C for 4 h. After that, 150 μL of lysate was added, and the absorbance was measured at 560 nm on a multifunctional enzyme marker.

### 4.14. Statistical Analyses

All data were expressed as mean ± SD of three independent experiments. Results were analysed using one-way analysis of variance (ANOVA). A *t*-test was used for each analysis, and *p* < 0.05 was considered a significant difference.

## Figures and Tables

**Table 1:** The list of primers.

Primers Name	Forward Primer (5′-3′)	Reversed Primer (5′-3′)
p53	CCAGATGACGCCTCCAGAGTG	TGAGAAGGGACAAAGGACGACAG
p38	ATCTCATTAACAGGATGCCAAGCC	CCAGCAAGTCAACAGCCAAGG
JNK	CCACCACCAAAGATACCTGACAAG	GGTTCTCTCCTCCAAGTCCATAAC
BAX	CATCTACCAAGAAGTTGAGCGAGTG	ACGGCTGCGATCATCCTCTG
Bcl-2	CGCAGAGGGGCTACGAGTG	CGGGCTGGGAGGAGAAGATG
GAPDH	GCGACTTCAACAGCAACTCCC	CACCCTGTTGCTGTAGCCGTA

## Data Availability

The data that support the findings of this study are available within the article.

## Supplementary Materials

The following supporting information can be downloaded at: https://www.mdpi.com/article/10.3390/ijms25042200/s1.
