# Correction: Li et al. Isolation and Identification of a Tibetan Pig Porcine Epidemic Diarrhoea Virus Strain and Its Biological Effects on IPEC-J2 Cells. *Int. J. Mol. Sci.* 2024, *25*, 2200

**DOI:** 10.3390/ijms252211855

**Published:** 2024-11-05

**Authors:** Mei Li, Meng Wang, Yao Xi, Shantong Qiu, Qiaoying Zeng, Yangyang Pan

**Affiliations:** 1 College of Veterinary Medicine, Gansu Agricultural University, Lanzhou 730070, China; lim@st.gsau.edu.cn (M.L.); wangmeng@gsau.edu.cn (M.W.); xy160117@163.com (Y.X.); qiushangtong1998@163.com (S.Q.); 2 Technology and Research Center of Gansu Province for Embryonic Engineering of Bovine and Sheep & Goat, Lanzhou 730070, China

There was an error in the original publication [1]: IPEC-J2 cell number error.

A correction has been made to Section 4, Materials and Methods, 4.1. Cells, first paragraph: IPEC-J2 cells (CVCL-2246) were grown in DMEM with 10% inactivated foetal bovine serum (FBS; PAN-Biotech, Aidenbach, Germany) and 1% penicillin/streptomycin (Invitrogen, Waltham, MA, USA) and incubated at 37 °C with 5% CO_2_ [52].

The authors state that the scientific conclusions are unaffected. This correction was approved by the Academic Editor. The original publication has also been updated.
