# Supplementary material for: Isolation and Identification of a Tibetan Pig Porcine Epidemic Diarrhoea Virus Strain and Its Biological Effects on IPEC-J2 Cells
Source: Int J Mol Sci. 2024 Feb 12;25(4):2200. doi: 10.3390/ijms25042200 (PMC10889329; doi:10.3390/ijms25042200)
Supplement: Supplementary file 1 [file ijms-25-02200-s001.zip › ijms-2854920-supplementary.pdf]

PEDV M gene

ATGCGAATTGACCCCCTCCCCAGTACTGTTATTGACGTATAAACGAAATATGTCTAACG
GTTCTATTCCCGTTGATGAGGTGATTGAACACCTTAGAAACTGGAATTTCACATGGAAT
ATCATACTGACGATACTACTTGTAGTGCTTCAGTATGGCCATTACAAGTACTCTGCGTTC
TTGTATGGTGTCAAGATGGCTATTCTATGGATACTTTGGCCTCTTGTGCTGGCACTTTCA
CTTTTTGATGCATGGGCTAGCTTTCAGGTCAACTGGGTCTTTTTTGCTTTCAGCATCCTT
ATGGCTTGCATCACTCTTATGCTGTGGATAATGTATTTTGTCAATAGCATTCGGTTGTGG
CGCAGGACACATTCTTGGTGGTCTTTCAATCCTGAAACAGACGCGCTTCTCACTACTTC
TGTGATGGGCCGACAGGTCTGCATTCCAGTGCTTGGAGCACCAACTGGTGTAACGCTA
ACACTCCTTAGTGGTACATTGCTTGTAGAGGGCTATAAGGTTGCTACTGGCGTACAGGT
AAGTCAATTACCTAATTTCGTCACAGTCGCCAAGGCCACTACAACAATTGTATATGGAC
GTGTTGGTCGTTCAGTCAATGCTTCATCTAGCACTGGTTGGGCTTTCTATGTCCGGTCA
AAACACGGCGACTACTCAGCTGTGAGTAATCCGAGTGCGGTTCTCACAGATAGTGAGA
AAGTGCTTCTAGTCTAAACAGAAACTTTATGGCTTCT

PEDV N gene

ATGCGAATTGACCCCCTCCCCAGTACTGTTATTGACGTATAAACGAAATATGTCTAACG
GTTCTATTCCCGTTGATGAGGTGATTGAACACCTTAGAAACTGGAATTTCACATGGAAT
ATCATACTGACGATACTACTTGTAGTGCTTCAGTATGGCCATTACAAGTACTCTGCGTTC
TTGTATGGTGTCAAGATGGCTATTCTATGGATACTTTGGCCTCTTGTGCTGGCACTTTCA
CTTTTTGATGCATGGGCTAGCTTTCAGGTCAACTGGGTCTTTTTTGCTTTCAGCATCCTT
ATGGCTTGCATCACTCTTATGCTGTGGATAATGTATTTTGTCAATAGCATTCGGTTGTGG
CGCAGGACACATTCTTGGTGGTCTTTCAATCCTGAAACAGACGCGCTTCTCACTACTTC
TGTGATGGGCCGACAGGTCTGCATTCCAGTGCTTGGAGCACCAACTGGTGTAACGCTA
ACACTCCTTAGTGGTACATTGCTTGTAGAGGGCTATAAGGTTGCTACTGGCGTACAGGT
AAGTCAATTACCTAATTTCGTCACAGTCGCCAAGGCCACTACAACAATTGTATATGGAC
GTGTTGGTCGTTCAGTCAATGCTTCATCTAGCACTGGTTGGGCTTTCTATGTCCGGTCA
AAACACGGCGACTACTCAGCTGTGAGTAATCCGAGTGCGGTTCTCACAGATAGTGAGA
AAGTGCTTCTAGTCTAAACAGAAACTTTATGGCTTCT

PEDV ORF3 gene

TTTCACCTTACGAAGCTTTTGAAAGGGTCCACGCGCAGTGATGTTTCTTGGACTTTTTC
AATACACGATTGACACAGTCGTCAAAGATGTCTCTAAGTCTGCCAACTTGTCTTCGGAT
GCTGTCCAAGAGTTGGAGCTTAATGTAGTTCCAATTAGACAAGCTTCAAATGTGACTG
GTTTTCTTTTCACCAGTGTTTTTATTTACTTCTTTGCACTGTTTAAAGCGTCTTCTTTGAG
GCGCAATTATGTTATGTTGGCAGCGCGTTTTGCTGTCATCTTTCTTTATTGCCCACTTTTA
TATTACTGTGGTGCATTTTTAGATGCAACTATTATCTGTTGCACACTTATTGGCAGGCTCT
TTTTAGTCTGCTTTTATTCCTGGCGCTATAAAAATGCGCTCTTTATTATCTTTAATACTACT
ACACTTTCTTTTCTCAATGGTAAAGCAGCTTATTATGACGGCAAATCCATTGTGATTCTA
GAAGGTGGTGACCATTACATCACTTTTGGCAACTCTTTCGTTGCTTTCGTTAGTAGCATT
AACTTGTATCTAGCTATACGTGGGCGGCAAGAAGCTGACCTACATCTGTTGCGAACTGT
TGAGCTTCTTGATGGCAAGAAGCTTTATGTCTTTTCGCAACATCATATTGTTGGCATTAC
TAATGCTGCATTTGACTCAATTCAACTAGACGAGTATGCTACAATTAGTGAATGATAATG
GTCTAGTAGTTAATGTTATACTTTGGCTTTTCGTACTCTTT

PEDV S gene

AAGGATCGATGTACTTTCACACTTAGCCTACCACAAGATGTCACCAGGTGCTCAGCTAA
CACTAATTTTAGGCGGTTCTTTTCAAAATTTAATGTTCAGGCGCCTGCAGTTGTTGTACT
GGGCGGTTATCTACCTATTGGTGAAAACCAGGGTGTCAATTCAACTTGGTACTGTGCTG
GCCAACATCCAACTGCTAGTGGCGTTCATGGTATCTTTCTTAGCCATATTAGAGGTGGTC
ATGGCTTTGAGATTGGCATTTCGCAAGAGCCTTTTGACCCTAGTGGTTACCAGCTTTATT
TACATAAGGCTACTAACGGTAACACTAATGCTACTGCGCGACTGCGTATTTGCCAGTTT
CCCAGCATTAAAACATTGGGCCCCACTGCTAATAATGATGTTACAACAGGTCGTAACTG
CCTATTTAACAAAGCCATCCCAGCTCATATGAGTGAACATAGTGTTGTCGGCATAACAT
GGGATAATGATCGTGTCACTGTCTTTTCTGACAAGATCTATCATTTTTATTTTAAAAATGA
TTGGTCCCGTGTTGCGACAAAGTGTTACAACAGTGGAGGTTGTGCTATGCAATATGTTT
ACGAACCCACTTACTACACTCTTAATGTTACTAGTGCTGGTGAGGATGGTATTTCTTATC
AACCCTGTACAGCTAATTGCATTGGTTATGCTGCCAATGTATTTGCTACTGAGCCCAATG
GCCACATACCAGAAGGTTTTAGTTTTAATAATTGGTTTCTTTTGTCCAATGATTCCACTT
TGGTGCATGGTAAGGTGGTTTCCAACCAACTCATTGTTGGTCAAATGTCTTTTGGCCAT
TCCTAAGATTTATGGACTAAGCCCATTTATTCTCCTTTAATCAAACATCGACGGTGTTTG
TAATGGAGCTGCTGTGCAGCGTGCAC
